# Plant Extracts as a Source of Natural Products with Potential Antimalarial Effects: An Update from 2018 to 2022

**DOI:** 10.3390/pharmaceutics15061638

**Published:** 2023-06-01

**Authors:** Giovane de Jesus Gomes Ribeiro, Sun Liu Rei Yan, Giuseppe Palmisano, Carsten Wrenger

**Affiliations:** 1 Unit for Drug Discovery, Department of Parasitology, Institute of Biomedical Sciences, University of São Paulo, São Paulo 05508-000, Brazil; 2 GlycoProteomics Laboratory, Department of Parasitology, Institute of Biomedical Sciences, University of São Paulo, São Paulo 05508-000, Brazil

**Keywords:** malaria, plant extracts, natural products, *Plasmodium*, ethnobotany, drug discovery

## Abstract

Malaria kills more than 500,000 people yearly, mainly affecting Africa and Southeast Asia. The disease is caused by the protozoan parasite from the genus *Plasmodium*, with *Plasmodium vivax* and *Plasmodium falciparum* being the main species that cause the disease in humans. Although substantial progress has been observed in malaria research in the last years, the threat of the spread of *Plasmodium* parasites persists. Artemisinin-resistant strains of this parasite have been reported mainly in Southeast Asia, highlighting the urgent need to develop more effective and safe antimalarial drugs. In this context, natural sources, mainly from flora, remain underexplored antimalarial spaces. The present mini-review explores this space focusing on plant extracts and some of their isolated natural products with at least in vitro antiplasmodial effects reported in the literature comprising the last five years (2018–2022).

## 1. Introduction

Malaria is still a worldwide public health problem, with an estimated 247 million cases and 619,000 deaths globally in 2022, principally affecting children in the African continent. Malaria primarily affects the intertropical region, where appropriate climatic and geographical conditions are suitable for the spread of the female *Anopheles* mosquito, the vector of the disease. The most prevalent species are *Plasmodium falciparum* and *Plasmodium vivax*, and the most severe form of the disease is caused by *P. falciparum* [1]. The rise of antimalarial resistance, mainly in Southeast Asia, due to the indiscriminate use of antimalarials and incomplete malaria drug treatment demonstrates the urgent need for the development of better and more efficacious drugs against the disease [2]. Although malaria prevention has shown considerable advances [3], the lack of an effective vaccine against the disease continues to increase the number of deaths in the African continent. In 2022, a ground-breaking malaria vaccine was prequalified by the WHO for the prevention of *P. falciparum* infections. This vaccine, named RTS, S/AS01 (Mosquirix) and developed in cooperation with the private pharmaceutical company GSK, was recommended by the WHO for the immunization of children from five months of age living in moderate- to high *P. falciparum* transmission regions in Africa. However, the effectiveness of this vaccine is not enough to completely halt the spread of severe malaria cases worldwide [4]. 

Despite the substantial public and private financial provision for malaria prevention resources and considerable scientific progress in the cellular, molecular, and clinical areas, chemotherapy remains a key tool for controlling the disease [5]. Malaria treatment has become increasingly complex, owing to the spread of artemisinin-resistant strains of *Plasmodium*. Recently, *P. falciparum* resistance to artemisinin derivatives has been detected in Southeast Asia [6,7]. As for *P. vivax*, the most important species on the African continent and in the Americas, there is a record of resistance to chloroquine and sulfadoxine-pyrimethamine in vivo and in vitro that has been increasingly documented [8,9].

Therefore, the current global malaria prevalence demonstrates the urgency for newer and more effective drugs against the disease [10]. Over the past millennium, different civilizations have used plants and their extracts for medicinal purposes. For example, the discovery of one of the most potent available antimalarials, artemisinin [11], was possible because of the almost 2000-year-old antimalarial recipe empirically used and written by ancient Chinese for traditional Chinese medicine. This demonstrates the importance of natural products and their derivatives for treating harmful human diseases.

Natural products have high structural diversity, biochemical specificity, and other molecular properties, making them promising lead compounds for developing new drugs [12].

Artemisinin was discovered in plant extracts (*Artemisia annua*); similarly, other antimalarials in therapeutics, such as quinine and quinidine, were first detected in the bark of the cinchona tree [13] more than 350 years ago in South America. The clear indication of promising antimalarials originated from plant extracts strongly suggests that new chemical entities may emerge from these valuable natural sources.

## 2. Malaria Scenario Worldwide: A Short Update

Malaria directly impacts communities’ health and economic development in tropical and subtropical regions. The World Health Organization (WHO) has estimated 247 million new cases in 2021. In 2021, 96% of malaria cases and deaths worldwide occurred in 29 of the 84 countries endemic to malaria (including the territory of French Guiana) [14]. Almost half of all cases came from four countries: Nigeria (26.6%), the Democratic Republic of the Congo (12.3%), Uganda (5.1%), and Mozambique (4.1%). Additionally, four countries accounted for just over half of all malaria deaths worldwide: Nigeria (31.3%), the Democratic Republic of the Congo (12.6%), the United Republic of Tanzania (4.1%), and Nigeria (3.9%). The percentage of total deaths from malaria among children under five years of age has decreased over the last 20 years, from 87.3% in 2000 to 76.8% in 2015; however, it has remained unchanged since then [15].

In 2021, the Southeast Asia region had nine countries where malaria is endemic, accounting for 5.4 million cases and contributing 2% of statistical malaria cases worldwide. India accounted for approximately 79% of all malaria cases worldwide in 2021. Approximately 40% of all cases in this region were caused by *P. vivax*. In the last 20 years, malaria cases have decreased by 76%, a reduction of about 17.4 million cases in 2021 [3]. An important recent achievement in the Asian continent was the inclusion of China as a certified malaria-free country.

In Latin America, Brazil, Colombia, and Venezuela are responsible for 79% of all malaria cases in the region. Most cases in this region are caused by *P. vivax* (71.5% in 2021).

The transmission of malaria in Brazil is restricted to the Legal Amazon, a territory that covers nine states: Acre, Amapá, Amazonas, Maranhão, Mato Grosso, Pará, Rondônia, Roraima, and Tocantins. This region accounts for approximately 99.7% of all malaria cases in the country. Two main species of *Plasmodium* are responsible for human malaria cases in Brazil: *P. vivax* and *P. falciparum*. Most reported cases were diagnosed as *P. vivax* infections, representing 83.0% (114,449) of registered cases, followed by *P. falciparum* infections, which accounted for 17.0% (23,408) of the cases [3].

As of 2020, epidemiological data indicate an annual decline in malaria cases, with 139,211 cases registered in 2021. The Legal Amazon is a territory where several indigenous communities live, including one of the largest indigenous groups, the Yanomami, who had approximately 11,530 cases of malaria in the last year, representing 9.93% of all malaria cases in Brazil [16]. However, there is a need for new interventions and improvements in disease surveillance and control.

There are also countries that achieved the WHO certification of malaria-free countries, including: Argentina (2019), El Salvador (2021), and Paraguay (2018), with zero cases of malaria for three consecutive years [17].

## 3. Methodology for Literature Selection and Previous Literature

For this mini-review, a database search was performed with Digital Science’s Dimensions platform (Dimensions AI), which also covers the PubMed database. The combination of keywords “*Plasmodium*” and “plant extract” in the title or abstract was used in the searches. The period covered is from 2018 to 2022. The search was initially conducted on 26 January 2023, but a few articles published after this date were also added due to the match of the keyword combination. The criteria for selecting the articles were primarily based on experimental data with a plant extract antiplasmodial activity less than 10 μg/mL. The articles were divided into two groups: those reporting natural products (divided into ethnobotanical studies and plant extracts) and those covering studies of isolated bioactive substances and possible pharmacological targets.

We have also analyzed recent reviews that referred to the use of medicinal plants as antimalarials [18,19,20,21,22,23]. They provide a thorough overview of the scientific literature with respect to the antimalarial effects of traditionally used medicinal plants, some of them mainly covering plants found in African countries, such as Ethiopia [20,22,23]. Another example is the review from Noronha and colleagues [21], which provides a broad revision of the literature concerning plants having antimalarial and antipyretic activity and the practical challenges of using these medicinal plants. In another review, Pan and collaborators [18] provide a comprehensive review covering more than 2000 plant extracts, including marine plants, and the discovery of 175 antimalarials from plants in the covered period of 2001–2017. The present mini-review differs from the cited previous literature, since it aims to provide an update on plant extracts and its most promising isolated compounds tested against *Plasmodium falciparum*, without restrictions to specific endemic plant species, and covering the last five years of scientific literature, as mentioned above.

## 4. Omics-Based Strategies for Targeted Isolation of Natural Products and Discovery of New Potential Drugs

Natural products play a key role in discovering new chemical entities [24], and plant extracts generally contain diverse chemical mixtures with different metabolites. Isolation of each chemical entity from these mixtures requires the use of combined analytical techniques, since the bioactive compounds are found in low concentrations and can also work in a synergistic mode. One challenging problem in the field of natural products is the identification of the mechanism of action (MOA) for small molecules. Target identification is crucial for compound optimization and further steps in the drug discovery process. However, it is also known that many drugs interact with different protein targets [25], which require broader target identification methods. In this sense, chemical proteomics may provide insights into the mechanism of action for small molecules. In chemical proteomics, the combination of chemistry, cellular biology, and mass spectrometry allows the generation of large data sets, with the possibility of understanding potential off-targets and how to generate a better lead compound. A crucial step in a chemical proteomics experiment is the probe design and synthesis, which depend on different probes, comprehensively revised by Chen and colleagues [25]. In chemical proteomics approaches, the natural products should be either chemically modified with an enrichment moiety (activity-based protein profiling) [26,27], linked to a resin, or conjugated with photoactivatable crosslinkers [28]. These approaches have been combined with quantitative label-free or label-based proteomics strategies to identify protein targets in complex biological systems. One drawback resides in the knowledge of structure–activity relationships, since chemical modifications can induce activity loss and binding to the wrong target.

The deconvolution of a protein target in an unbiased way has also been achieved using other approaches that explore protein stability upon small molecule binding. Protein stability can be challenged by raising the temperature, which will induce denaturation, aggregation, and precipitation. Proteins binding to a specific small molecule will respond differently to temperature changes. Thermal Proteome Profiling (TPP), cellular thermal shift assay coupled with mass spectrometry (CETSA^®^) [29], and Proteome Integral Solubility Alteration (PISA) [30] are based on thermal shifts. These assays can be performed in vitro, intact cell lines or cell lysates, and in vivo, in tissues and biofluids. MS-CETSA was applied to identify *P. falciparum* purine nucleoside phosphorylase as the target of quinine [31]. Another assay, termed drug affinity responsive target stability (DARTS), measures the resistance of a protein to hydrolysis by a broad specificity protease in the presence of a known small molecule [32]. The same principle was exploited in the limited proteolysis/LiP-small molecule mapping (LiP-SMap) [33]. Protein stability upon interaction with a small protein was also probed using the rate of methionine oxidation upon incubation with a denaturing agent. This assay was termed stability of proteins from rates of oxidation (SPROX) [34]. Recently, the resistance of a protein binding to a small molecule was probed using organic solvents. In this assay, termed solvent proteome profiling (SPP), the association of proteins with small molecule makes them more or less susceptible to solvent-induced denaturation [35]. The small molecule does not need to be modified in these assays, and its target(s) can be identified using a purified protein or a complex proteome. One drawback of these strategies is the solubility of membrane proteins. Indeed, the need to work with soluble proteins in their native state limits their applicability to multipass transmembrane proteins. Combining mass spectrometry-based proteomics strategies with these assays allows the identification of unknown targets.

Metabolomics has evolved to allow the analysis of thousands of metabolites from natural extracts [12], and it has been used to characterize metabolites of less than 1.5 kDa, such as amino acids and lipids, based mainly on nuclear magnetic resonance (NMR) and mass spectrometry (MS) data. It has already been used for the identification of drug targets, as well: early metabolomics studies permitted, for example, the development of FDA-approved drugs enasidenib (Idhifa^®^) and ivosidenib (Tibsovo^®^) for the treatment of acute myeloid leukemia [36,37,38,39].

Moreover, the application of metabolomics in the screening of natural product extracts via hyphenated techniques allows detailed structural characterization [40]. It also permits implementing databases that can be used to discover new bioactive natural products [41].

**Figure 1:**
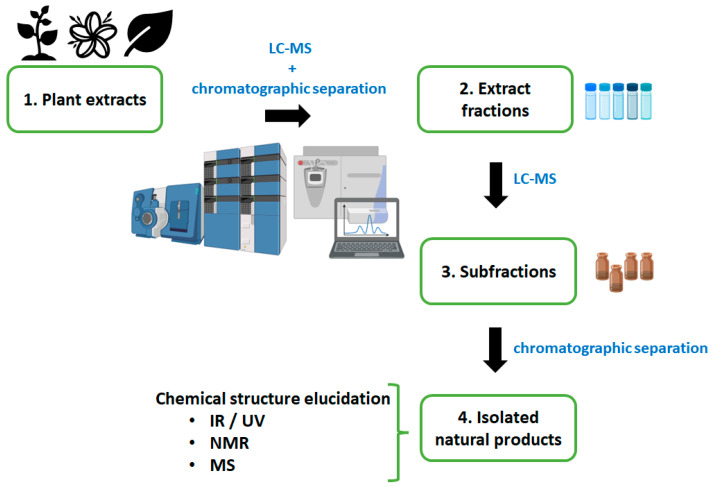
General workflow for discovery and isolation of chemical compounds derived from natural products. The figure was generated with the help of BioRender.com (License #2364–1511, Toronto, ON, Canada).

Recent developments in analytical chemistry include dereplication and high content screening (HCS). Dereplication refers to a process for the fast identification of known secondary metabolites in natural products, in which MS-based methods are key to the success of that process [42,43,44], and HCS methods are those that combine automated microscopy with living cell-based assays [45,46,47,48], which allows, e.g., the screening of plant extract libraries and subsequent selection of the best hits based on the phenotypic screening results. However, the isolation of chemical compounds from crude natural extracts remains a major challenge in the field [42]. The extraction method determines the chemical compound classes present in the purified fractions. Based on the polarity of the molecules present in the extract, using different organic solvents can selectively isolate a range of different compounds, directly affecting phytochemical isolation and yield. The combination of solvents would be a strategy to improve the extraction of compounds with different polarities [49], as illustrated in Figure 1. After identifying a crude extract with promising pharmacological activity, the extract is fractionated and guided by bioactivity until pure bioactive compounds are isolated [50].

Other limitations of crude extraction steps include the presence of known natural product molecules, chemical compounds without drug-like characteristics, and insufficient amounts of natural products for characterization [47]. These technical challenges can be addressed by the development of new methods using dereplication, extraction, and prefractionation of extracts [51]. Another approach is the use of metabolomics and genomics, in which data generated by molecular networks are analyzed and common characteristics of the natural extract composition are determined. The low yield in the extraction process and the structural elucidation of the chemical compounds are other aspects to be addressed in the field [47]. Bioinformatics tools and genomic engineering may also be helpful. Tandem mass spectrometry (MS/MS) libraries allow fast, reproducible, and accurate analysis of thousands of MS/MS spectra. Because there is a need for reliable information on MS/MS, different databases, including NIST, MassBank, MoNA European MassBank, LipidBlast, Wiley MSforID, and METLIN, are available for this purpose. In addition, the web platform Global Natural Product Social Molecular Networking allows the generation of a molecular network in which molecules are grouped based on their fragmentation pattern of similarity and similarity in the identification of metabolites [52].

## 5. Novel Antimalarials Derived from Plant Extracts and Isolated Natural Products

**Figure 2:**
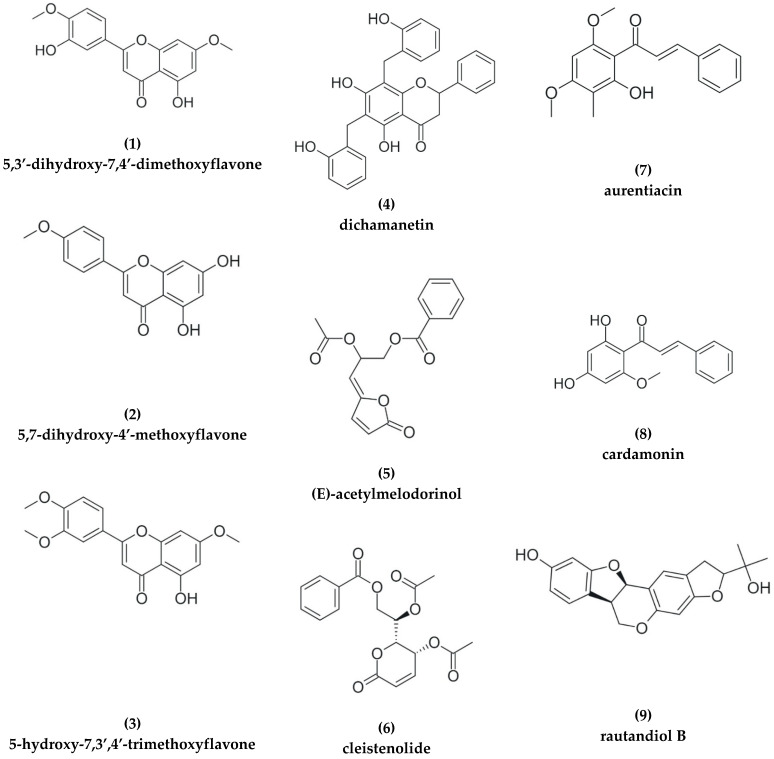

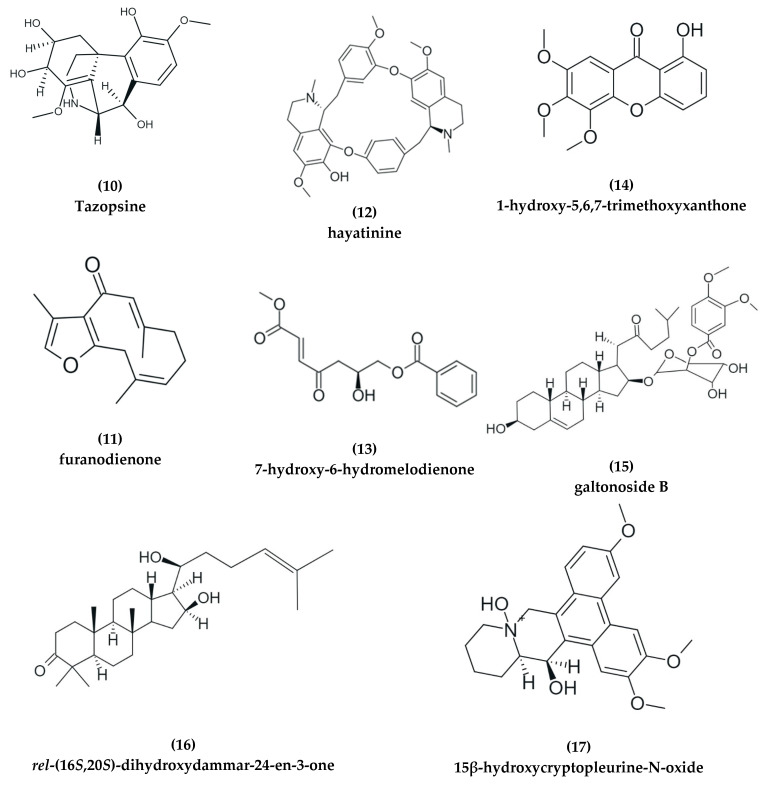

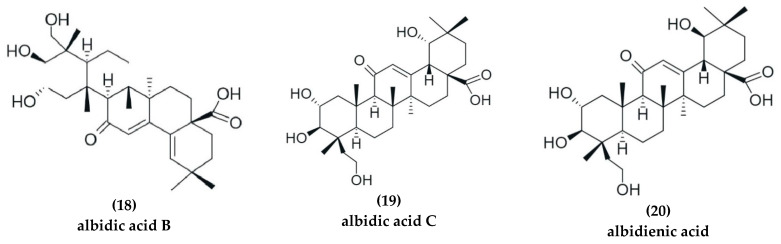
Isolated and characterized chemical compounds from different plant sources that showed low IC_50_ (<10 μM).

Antiplasmodial natural products are organized into families of promising extracts, and isolated compounds are organized in classes, such as alkaloids, terpenes, and polyphenols, with subclasses when applicable. The in vitro antiplasmodial activities described in this review were determined using various strains of *P. falciparum* with different drug sensitivities. The chloroquine-sensitive strains were 3D7, NF54, D6, HB3, D10, and TM4/8.2. Other strains used were the chloroquine-resistant strains Dd2, FcB1, and Pf INDO, as well as the multiresistant strains K1, W2, FCR3, and K1CB1. Different assays were used to determine in vitro antiplasmodial activity of the compounds. The most cited methods are the radioactive hypoxanthine incorporation assay, the colorimetric enzyme-linked immunosorbent assay (ELISA) that measures *P. falciparum* lactate protein dehydrogenase (pLDH), DNA-based fluorometric method using Pico Green Assay (SYBR), Malstat reagent, and microscopy. Regardless of the in vitro assay method, in this review, the inhibitory concentration range and extracts with half maximal inhibitory concentrations (IC_50_) ≤ 10 µg/mL were considered active [53]. Only isolated compounds with IC_50_ ≤ 10.0 µM were considered for further studies [54,55,56]. Based on these criteria, from the selected literature, a total of twenty isolated natural products derived from three different plant species were found to inhibit *Plasmodium* parasite proliferation in vitro (Table 1 and Figure 2).

Phytochemical investigation of aqueous and ethanolic leaf extracts of *Azadirachta indica* (Meliaceae) showed inhibition of *P. falciparum* with IC_50_ values of 7.4 µg/mL and 8.6 µg/mL, respectively. A study on the in vitro antimalarial activity of extracts from indigenous plant species in Kebbi State justifies the traditional use of the plant in malaria treatment, and further research may help identify and characterize the active principles of these plants [57].

The *Homalolepis suffruticosa* of the Simaroubaceae family is known for its popular use in treating malaria. *Homalolepis Turcz*. is a neotropical genus named Quinquina de Cayenne and represents one of the many drugs called ‘falsa quina’ (fake quine), which is used to treat malaria in South and Central America. In a bioassay-guided assay [58], the methanol extract of the roots of *H. suffruticosa* showed high in vitro antiplasmodial activity with an IC_50_ of 1.88 μg/mL in moderate cytotoxic HepG2 (CC_50_ = 41.93 μg/mL) and an SI = 22.30.

*Goniothalamus lanceolatus* (Annonaceae), an endemic plant from Borneo, is traditionally used in Malaysia to alleviate fever, as part of antimalarial treatment by folk medicine. Upon screening, methanol extract from roots exhibited potent antiplasmodial activity against both *P. falciparum* 3D7 (IC_50_ = 2.7 μg/mL, SI = 140) and K1 strains (IC_50_ = 1.7 μg/mL, SI = 236) [59]. The Annonaceae family is the largest family in Magnoliales order, comprising more than 100 genera and about 2400 species [76]. The large number of genus/species, with some of them reported in the literature as having antimalarial effects, such as those mentioned above for *Goniothalamus lanceolatus,* may also suggest further thorough investigation of potential antimalarials in that family [77].

According to a study by Dwivedi and collaborators [60], the plant *Vitex negundo* (Lamiaceae) is traditionally used for the treatment of malaria by indigenous tribes in certain countries, such as India and Malaysia. In vitro studies have reported the antimalarial use of the plant in traditional medicinal systems. The active extract of *V. negundo* leaf exhibited antimalarial activity with IC_50_ values of 7.21 μg/mL and 7.43 μg/mL against strains 3D7 and K1, respectively.

*Petasites japonicus*, an herb of the family Asteraceae, has been used to treat several human diseases. In Asian countries, the traditional use of *P. japonicus* is based on its many recognized pharmacological activities, including analgesic, anti-inflammatory, bactericidal, and antimalarial properties. A phytochemical study showed that the ethanolic extract from *P. japonicus* leaves significantly inhibited the parasite with IC_50_ values of 8.48 μg/mL and 7.83 μg/mL against strains 3D7 (CQ-sensitive) and Dd2, respectively [61]. The antiplasmodial activity of *P. japonicus* was also tested in vivo with *P. berghei* ANKA infected mice. The ethanolic extract of *P. japonicus* in a dose of 200 mg/kg lowered the parasitemia after four days of infection with a decrease percentage in the range from 65.21% to 97.14% [61].

In another study, the medicinal plant *Senna occidentalis* was evaluated for its phytochemical and activity against *P. falciparum* 3D7. The methanol extracts of the leaves and seeds of *S. occidentalis* (L.) (Fabaceae) (Leguminosae) showed significant activity against *P. falciparum* 3D7 with IC_50_ values of 12.19 µg/mL and 6.82 µg/mL, respectively [62]. In Kenya, the roots of the plant *S. occidentalis* are traditionally used for malaria treatment by the Luhya, Digo, and Duruma communities: the roots of the plant are decocted and taken orally three times a day over the course of three to four days. The methanolic extracts of *S. occidentalis* show antiplasmodial activity with a calculated IC_50_ of 1.76 μg/mL against 3D7 strain. No cytotoxicity was observed against a Vero cell line (CC_50_ 247, IS of 140). The methanolic extract was also tested in vivo (*P. berghei* ANKA), showing an antiplasmodial effect of 200 mg/kg and effective dose (DE_50_) of 34.13 ± 5.14 mg/kg [78].

Plants of the genus *Nauclea* (Rubiaceae) are frequently used as folk medicines for the treatment of various diseases. Antimalarial activity of the hexane (IC_50_ = 1.93 μg/mL) and methanol (IC_50_ = 3.91 μg/ mL) fractions of *Nauclea orientalis* leaves against 3D7 *P. falciparum* was observed [63].

According to a previous investigation [64], the aqueous extracts of the leaves of the antimalarial *Alchornea cordifolia* (Euphorbiaceae) exhibited moderate activities against NF54 (chloroquine-sensitive parasite; IC_50_ of 5.8 μg/mL), CamWT_C580Y (artemisinin-sensitive parasite; IC_50_ 17.8 μg/mL), and IPC 4912 (artemisinin-resistant parasite; IC_50_ 15.8 μg/mL). The results of this study support the traditional use of the species *A. cordifolia* in Ghana as a folk remedy for treating malaria.

The *Helianthus annuus* (Asteraceae) plant is traditionally used as a medicinal remedy for several diseases, including malaria, in Oro-oro Ombo, Malang, East Java, and Indonesia. The ethanol extract of the roots and leaves of the species *H. annuus* exhibited antimalarial activity, with IC_50_ values of 2.3 and 4.3 μg/mL, respectively, against *P. falciparum* 3D7 [65]. The in vivo antiplasmodial effects of the ethanolic extract from *H. annuus* were also evaluated: 100 mg/kg with DE_50_ of 63.6 ± 8.0 mg/kg and 59.3 ± 13.2 mg/kg [65].

In Côte d’Ivoire, several traditional pharmacopeia plants, such as *Pericopsis laxiflora* and *Harungana madagascariensis,* have been used by various traditional practitioners for malaria treatment. The aqueous extract of the bark of *H. madagascariensis* (Hipperiacaceae) showed antiplasmodial activity, with an IC_50_ value of 6.16 µg/mL against *P. falciparum* NF54 [66]. The methanolic extracts of *P. laxiflora* (Euphorbiaceae) showed the best antiplasmodial activity, with an IC_50_ value of 7.44 µg/ mL against K1 [66].

*Pleiocarpa*, which belongs to the Apocynaceae family, is native to tropical Africa and is distributed from Senegal to Tanzania and Zimbabwe. Medicinal plants of this genus are well known for their use in the traditional treatment of pain, fever, and malaria. A study [67] investigated the dichloromethane/methanol (1:1) extract of *Pleiocarpa bicarpellata* roots, which showed strong antiplasmodial activity (IC_50_ = 3.5 µg/mL) against *P. falciparum* NF54. Negative cytotoxicity against L6 cells was observed.

Another recent study [58] on the *Psychotria* plant, which is native to Amazonia and Atlantic Forest biomes in Brazil, evaluated the antiplasmodial effect of the acid–base extracts of *Psychotria apoda* and *P. colorata* (Rubiaceae), which showed 90% inhibition of the parasite *P. falciparum* W2.

*Andrographis paniculata* is a tropical medicinal plant belonging to the Acanthaceae family. It is traditionally used for medicinal purposes in Hong Kong, Philippines, Taiwan, China, India, Malaysia, Indonesia, and many South Asian countries. The chloroform extract of whole plants of *Andrographis paniculata* exhibited antimalarial activity with IC_50_ values of 6.36 μg/mL and 5.24 μg/mL against strains 3D7 and K1, respectively, with no evidence of significant cytotoxicity against a mammalian cell line (CC_50_ > 100 μg/mL) [69].

Chaniad and collaborators [70] investigated the plant *Globba malaccensis* (Zingiberaceae), which is native to Nakhon Si Thammarat Province, Thailand. The ethanolic extract of *G. malaccensis* rhizomes showed promising antimalarial activity against *P. falciparum* K1 (IC_50_ = 1.50 µg/mL), with less toxicity to Vero cells (CC_50_ > 80 µg/mL). The ethanolic extract of this plant exhibited a considerable reduction in *P. berghei* parasitemia (murine model). A maximum suppressive effect of this extract (60.53%) was observed with the highest administered dose (600 mg/kg).

In Indonesia, the plant *Sanchus arvensis* L. (Asteraceae) is a traditional medicinal plant employed for malaria treatment. The ethyl acetate fraction obtained from leaves of *S. arvensis* showed in vitro antiplasmodial activity, with an IC_50_ of 2.92 μg/mL (3D7 strain), and it did not show cytotoxicity against human hepatic cells (Huh7it-1), with an SI of 97.03 [71]. The fraction was also tested in vivo (mice infected with *P. berghei* ANKA), showing an antiplasmodial activity below 100 mg/kg/day and ED_50_ 46.31 ± 9.36 mg/kg. Chloroquine and chloroquine diphosphate were the positive controls for the in vitro studies, and DMSO served as the negative control.

The plant *Terminalia arjuna* (Roxb. ex DC.) (Wight and Arn.), known as “arjuna” or “Sa-mor-tes” in Thailand, has been traditionally used for various human diseases, given its antibacterial, antitumoral, and antiallergic effects. Against *P. falciparum* (strain K1), in vitro results showed an IC_50_ of 4.05 μg/mL [72]. In Vero cells, the CC_50_ was higher than 200 μg/mL, an indication of low cytotoxicity. For the in vitro study, the authors employed artesunate as a positive control and DMSO as a negative control. The in vivo study with aqueous extract of the *T. arjuana* fruits demonstrated a suppression of 67.95% of *Plasmodium* parasites at a dose of 600 mg/kg (*P. berghei* ANKA, murine model) [72]. Another plant species from the same genus, *Terminalia bentzoe* (L.) L.f. (Combretaceae) [73], was tested against *P. falciparum* (Dd2 strain), with an IC_50_ of 6.7 μg/mL and low cytotoxicity in Vero cells and CC_50_ 207.03 μg/mL.

Another plant with potential antimalarial properties is *Bridelia atroviridis* Müll. Arg., a bush from Euphorbiaceae family that is found in equatorial Africa. This plant has been used in traditional medicine to treat various diseases, such as diabetes, gonorrhea, and fungal infections. Djouwoung and colleagues [74] have shown the antiplasmodial activity of hydroethanolic bark extract of *Bridelia atroviridis* Müll. Arg., exhibiting an in vitro antiplasmodial activity, with an IC_50_ of 8.08 μg/mL against the Dd2 strain. It also presented low cytotoxicity (CC_50_ > 100 μg/mL). The in vivo tests were performed in *P. berghei* NK 65 infected rats, resulting in a calculated ED_50_ of 89 mg/kg.

In Nigeria, the plant species *Annickia affinis* (Exell) (Versteegh and Sosef) from Annonaceae family is traditionally used for the treatment of fever and malaria. Decoction with water for this plant resulted in IC_50_ 1.49 μg/mL (3D7 strain), while the CC_50_ was above 200 μg/mL [75]. In the in vivo test of infected mice with *P. berghei* ANKA, the *A. affinis* methanol extract at an oral dose of 100 mg/kg suppressed parasite growth by 32.46%. It was also investigated whether the plant has synergism with other plants as well, such as secondary metabolites from the mixture of plants named hepta-herbal *Agbo-iba* [75].

In another recent study, Ledoux and colleagues [79] investigated the potential antimalarial effects of the leaves of *Casearia coriacea* Vent. (Salicaceae), a plant endemic to the Mascarene Islands. Clerodane diterpenes were isolated from dichloromethane fraction and tested against the *Plasmodium* parasite: caseamembrin T, corymbulosine I, and isocaseamembrin E, with IC_50_ values of 0.25 µg/mL, 0.40 µg/mL, and 0.51 µg/mL, respectively. Cytotoxicity activity was considered slightly selective for pancreatic carcinoma (PANC-1). Structural determination was performed using NMR and mass spectrometry. This article is the first report of the isolation of these compounds from *C. coriacea* and their antiparasitic potentials. Due to the possible action of compound corymbulosine in apoptotic death, the compound could have its studies extended to cancer, as well. An interesting approach in this article is the fact that the authors have employed the *zebrafish* model as in vivo cytotoxicity assay rather than using mice model. Yet, the adjustment of the zebrafish model to test phytochemical compounds is also an open field.

## 6. Alkaloids: Potential Antimalarial Activity

Bioassay-guided fractionation of *Hypoestes forskaolii* (Vahl) R.Br. produced a phenanthroquinolizidine alkaloid, the isolated compound 15β-hydroxycryptopleurine-N-oxide (**17**), which has antiplasmodial activity. The chloroquine-resistant (K1) and chloroquine-sensitive (FCR3) *P. falciparum* strains were very sensitive to the antimalarial activity of 15β-hydroxycryptopleurine-N oxide, with IC_50_ values of 6.11 and 5.13 nM, respectively, while using chloroquine as the positive control [80]. A total of 15 mg was obtained from the plant, and a combination of techniques were used to purify the compound: silica gel column chromatography, high performance liquid chromatography (HPLC), and NMR (H, C, and two-dimensional).

From *Cissampelos pareira* (Linn) (Menispermaceae), a plant traditionally used to treat various diseases in India, a root ethyl acetate fraction with the most promising compound hayatinine (**12**), a bisbenzylisoquinoline alkaloid, was isolated (a total of 23 mg), having IC_50_ values of 0.41 μM (INDO) and 0.509 μM (3D7) [81]. Chloroquine was used as the positive control. Silica gel column chromatography, HPLC, NMR (H, C, and two-dimensional), and liquid chromatography coupled to mass spectrometry (LC/MS) were utilized for this alkaloid.

The phytochemical investigation of *Aconitum heterophyllum* (Wall. ex Royle) (Ranunculaceae) roots led to the identification of 2-*O*-cinnamoyl hetisine, a diterpenoid alkaloid in the chloroform fraction of the plant [82]. Antiplasmodial in vitro studies indicated an IC_50_ of 1.92 μM (INDO) and an IC_50_ 10.8 μM (3D7). The yield of this compound was very low: from 2.6 g of chloroform fraction, only 16 mg of this hetisine were obtained.

The tazopsine alkaloid (compound **10** in Figure 2), isolated from *Strychnopsis thouarsii* Baill. (Menispermaceae), an endemic Malagasy plant, showed antiplasmodial activity and an of IC_50,_ 7.88 µM against the NF54 strain, as well as a value of 4.07 µM against the 3D7 strain [83]. However, in *P. yoelii* infected mice, it was considered non-prophylactic, with 70% protection at a 100 mg/kg dose.

## 7. Terpenoids: Potential Antimalarial Activity

Sesquiterpene lactones have emerged as good starting points in the search for transmission-blocking drugs, with several studies demonstrating their potential. The most widely investigated member of this class of compounds is artemisinin, along with its derivatives. Greve and collaborators reported [84] that a triterpene derivative, compound *rel*-(16*S*,20*S*)-dihydroxydammar-24-en-3-one (**16**), had the most promising antimalarial activity. For this triterpene derivative, silica gel column chromatography, HPLC, NMR (H, C, and two-dimensional), and LC/MS were used.

The dichloromethane extract obtained from *Commiphora myrrha* Engl. (Burseraceae), the oleo-gum resin, showed promising in vitro activity against *P. falciparum* NF54, with an IC_50_ value of 1 µg/mL. Bioactivity-guided fractionation led to the isolation and characterization of 18 sesquiterpenoids. The sesquiterpene furanodienone (**11**) and the triterpene *rel*-(16*S*,20*S*)-dihydroxydammar-24-en-3-one (**16**) were the most active compounds found in this study, with IC_50_ values of 7.4 and 2.8 µM, respectively, with chloroquine as the positive control [84]. The techniques employed to isolate the compounds were silica gel column chromatography, HPLC, flash chromatography, NMR (H, C, and two-dimensional), and LC/MS.

Phytochemical investigation of the n-buthanolic extract of the roots of *Terminalia albida* (Sc. Elliot) (Combretaceae) by Balde and collaborators [85] led to the isolation and identification of ten oleanane triterpenoids, of which albidic acid B (**18**), albidic acid C (**19**), and albidienic acid (**20**) showed moderate antiplasmodial activity, with IC_50_ values ranging in the interval between 6 μM and 7 μM against K1 strain, also with chloroquine as the positive control. The isolation of the compounds was possible by employing the techniques of silica gel column chromatography, HPLC, NMR (H, C, and two-dimensional), and LC/MS.

The ethanolic extract of *Cleistochlamys kirkii* (Benth.) (Oliv.) (Annonaceae) root bark showed a 72% inhibition against chloroquine-sensitive 3D7-strain malarial parasite *P. falciparum*. The monotypic genus *Cleistochlamys* has a geographically restricted distribution in Southern Africa, and traditional uses of the plant comprise treatment of tuberculosis and rheumatism [86]. The isolated metabolites dichamanetin (**4**), (E)-acetylmelodorinol (**5**), and cleistenolide (**6**) showed IC_50_ values of 9.3, 7.6, and 15.2 µM, respectively, against *P. falciparum* 3D7, with chloroquine and artesunate as positive controls. Both the extract and the isolated compounds exhibited cytotoxicity against the negative aggressive breast cancer cell line MDA-MB-231 [86]. Different techniques were used to isolate the compounds: silica gel column chromatography, Sephadex LH 20, HPLC, NMR, infrared spectroscopy (IR), and tandem mass spectrometry (MS/MS).

Kim and colleagues [87] reported eight diterpenoids from the whole plant of *Vitex rotundifolia* L. f. (Verbenaceae). It is an important medicinal plant distributed in Korea, Japan, India, and China. The traditional uses of the plant comprise gastrointestinal diseases, chronic bronchitis, and migraine. The antiplasmodial activity was tested against the Dd2 strain, with an IC_50_ of 1.2 μM for the compound named abieta-11(12)-ene-9β,13β-endoperoxide, which was obtained from the *n*-hexane fraction (5.1 g), yielding a total of 15.4 mg. The β-peroxy bridge in this compound shows its importance for the antiplasmodial activity (Dd2), increasing it by up to 54 folds. Another promising isolated compound was Vitetrifolin E, with an IC_50_ 1.3 μM, which was isolated with a yield of 55.7 mg from 21 g of the methanolic fraction.

Two compounds isolated from the stem barks of *Trichilia monadelpha* (Thonn.) (J. J. De Wilde) (Meliaceae) were very active against the *P. falciparum* 3D7 strain: grandifotane A showed an IC_50_ of 1.37 µM and khayanolide D an IC_50_ of 1.68 µM, both against the 3D7 strain [88]. Both isolated compounds were obtained in the *n*-hexane/ethyl acetate fraction (150 mg for grandifotane A and 15 mg, both from initial 150 g of extract). The genus *Trichilia* comprises about 70 species distributed in tropical regions, mainly Africa and America, and the species *T. monadelpha* is used in traditional medicine for treating a variety of diseases, including depression, epilepsy, syphilis, and rheumatism [88].

Another genus of plants, *Croton*, is used in Tanzanian traditional medicine for the treatment of infections, malaria, tuberculosis, and cancer. In a recent study [89], stem bark and leaf extracts of *Croton kilwae* Radcl.-Sm. (Euphorbiaceae) endemic species (Tanzania and Mozambique) yielded the isolation of three new crotofolane diterpenoids with antiplasmodial activity at 50 μM concentration, and which were named as crotokilwaepoxides. Artesunate at 5 μM concentration was utilized as positive control and drug vehicle DMSO as negative control in parasitaemia assays. The structure of the antiplasmodial compounds was determined via NMR spectroscopic data and mass spectrometry, and one compound was identified as a new crotofolane 9-methoxycrotokilwaepoxide A.

Still, in the terpenoids class, dimeric sesquiterpenoids isolated and characterized from *Sarcandra glabra subsp. Brachystachys* (Blume) (Verdc.), a plant species of tropical biome in south Asia and a long-known folk medicine in China, may also look promising in terms of antimalarial properties. Zhou and collaborators have isolated and characterized many compounds of this chemical class from plants of Chinese folk medicine [90], and, recently, it was found that one of the isolated dimeric sesquiterpenoids showed an EC_50_ value in the picomolar range (4.3 pM) [91], testing in vitro against *P. falciparum* Dd2 with DMSO as negative control. The in vitro compound has about 1000-fold potency compared to artemisinin. Therefore, this chemical class may be of particular interest in malaria drug discovery.

## 8. Polyphenols: Potential Antimalarial Activity

The plant *Sphaerocoryne gracilis* (Oliv. ex Engl. and Diels) (Verdc.) (Annonaceae) found in Tanzania was screened for antiplasmodial activity against chloroquine-sensitive (3D7) and chloroquine-resistant (Dd2) strains of *P. falciparum*. Compounds showing promising antiplasmodial activities, namely, (Z)-Sphaerodiol, (Z)-acetylmelodorinol, 7-hydroxy-6-hydromelodienone (**13**), and dichamanetin, inhibited the proliferation of *P. falciparum* (3D7 and Dd2), with IC_50_ values of 1.4–10.5 μM [92]. Silica gel column chromatography, Sephadex LH 20, HPLC, NMR (one-dimensional and two-dimensional) were employed for the isolation of compound (**13**). The genus *Sphaerocoryne* consists of only three species, namely, *S. fruticosum*, *S. gracilis* (ssp. *Engleriana* and *gracilis*), and *S. punctulatum*, which are native to some countries of the African continent, e.g., Angola, Zambia, Mozambique, and Tanzania [92].

Du and collaborators [93] performed a bioassay-guided isolation of the methanol extract of *Galtonia regalis* (Hilliard) (B.L. Burtt) (Asparagaceae), and the extract exhibited antiplasmodial activity with IC_50_ value less than 1.25 μg/mL. Two known cholestane glycosides and five new cholestane glycoside galtonosides were isolated after bioassay-guided fractionation. Galtonoside B (**15**) showed the most potent antiplasmodial activity, with an IC_50_ value of 214 nM against the multidrug-resistant Dd2 strain of *P. falciparum*. Silica gel column chromatography, Sephadex LH-20, HPLC, NMR (one-dimensional and two-dimensional), and MS/MS were used in combination for the isolation of the compound.

Phytochemical investigation revealed that 5,3′-dihydroxy-7,4′-dimethoxyflavone (**1**), 5,7-dihydroxy-4′-methoxyflavone (**2**), and 5-hydroxy-7,3′,4′-trimethoxyflavone (**3**) had strong antiplasmodium activity against the 3D7 strain of *P. falciparum,* with IC_50_ values of 2 nM, 5 nM, and 1 nM, respectively. The compounds were isolated from the stem bark of *Drymis beccariana* Gibbs (Winteraceae) and indicate a potential source of antimalarial agents [94]. Silica gel chromatography, HPLC, ultraviolet-visible spectroscopy (UV), IR, NMR, and MS/MS were used for compound isolation.

A biodereplication workflow was applied to an ethanolic extract of the medicinal plant *Piper coruscans* Kunth (Piperaceae) against the *Plasmodium falciparum* strain 3D7. This resulted in the isolation of two antimalarial chalcones, aurentiacin (**7**) and cardamonin (**8**), with IC_50_ values of 2.25 and 5.5 µM, respectively [95]. The combination of NMR, MS/MS and LC/MS allowed isolation and characterization of the compounds.

Dawurung and collaborators [96] identified a lead compound with a pterocarpene structure in the plant *Neorautanenia mitis* (A. Rich) Verdc. (Fabacae), which was developed into a drug against *P. falciparum*, Rautandiol B (**9**), with IC_50_ values of 400 nM (TM4/8.2) and 740 nM (K1CB1). As with the above-mentioned studies, spectroscopy techniques were utilized: UV, IR, and chromatographic techniques HPLC, Sephadex LH 20, silica gel column, and MS/MS.

In another study, the plant *Aloe marlothii* A.Berger (Xanthorrhoeaceae), found in southern African countries, and traditionally used in folk medicine to treat different diseases, was tested against asexual and sexual forms of *P. falciparum* parasites. Mianda and collaborators [97] isolated six different compounds of this plant, and the most promising compound identified was aloesaponarin I, with an IC_50_ of 1.54 μg/mL (5 μM) against NF54 strain and IC_50_ of 1.58 μg/mL (5 μM) against K1 strain.

In a recent study [98], a plant found in Asia, named *Putranjiva roxburghii* Wall. (Putranjivaceae), was found to contain the polyphenol quebrachitol, which had an IC_50_ of 0.87 μg/mL and at a dosage of 30 mg/kg indicated a chemosuppression of 73.26% in a murine model (*P. berghei* K173). High selectivity index was obtained for this compound (SI = 158).

The species *Paeonia officinalis* L. (Paeoniaceae) belongs to a genus that comprises about 33 species. They are distributed in Europe and Asia, and 11 species are found in China. The roots of *P. officinalis* are used in traditional Chinese medicine due to its anti-inflammatory, analgesic, and sedative properties, as well as its antihypertensive and anti-ulcer properties. Through the combination of chromatographic techniques, including Sephadex LH-20, the compounds identified as methyl gallate and galloyl paeoniflorin were isolated and tested in vitro against two different strains of *P. falciparum* (D6 and W2) [48], which provided better IC_50_ values against the W2 strain—0.61 and 2.91 µg/mL, respectively.

Antimalarial compounds from *Mammea siamensis* (Miq.) (T. Anders.) (Calophyllaceae) flowers, which were isolated via a bioassay-guided workflow, exhibited antimalarial activity. The result suggested that 1-hydroxy-5,6,7-trimethoxyxanthone (**14**) is a possible lead structure and a potent inhibitor of the *Pf*LDH enzyme. Negative cytotoxicity in Vero and HepG2 cells was observed [70]. Techniques, such as silica column gel, HPLC, and NMR (one-dimensional and two-dimensional) were employed to isolate the antimalarial structure.

## 9. Conclusions and Future Perspectives

The search for antimalarial compounds isolated from plant extracts remains challenging. The benefits of searching such compounds in flora are evident: the plethora of chemical structures potentially isolated from natural sources is still an underexplored space, and the diversity in the traditional use of endemic plants also reinforces the need for isolation of possible antimalarial compounds. However, different aspects in plant extracts and their isolated compounds are crucial to be understood: the possible synergisms that exist when combining plant extracts through different techniques, the presence of factors that interfere in the composition of the extracts such as seasonality that may interfere in the levels of plants secondary metabolites, and the empirical evidence originated from the traditional use.

This mini review has provided an overview of the last five years of the literature regarding plant extracts and their isolated natural compounds with potential antimalarial effects. Twenty chemical compounds isolated from plants with IC_50_ < 10 μM against at least one *P. falciparum* strain were selected and discussed. Several rich antiplasmodial fractions derived from plant extracts have been reported in the literature. However, the link between traditional knowledge and further research in discovering potential natural products with pharmacological properties must be reinforced, and hyphenated techniques are crucial for better characterizing the chemical compounds structures and their targets.

In particular, a closer connection with the local communities and better documentation of the local scientific information gathered is needed to further aid natural products research. Some families, such as Annonaceae, Meliaceae, Asteraceae, and Euphorbiaceae, are rich sources of bioactive compounds capable of inhibiting *Plasmodium in vitro*. More specifically, the families Annonaceae and Asteraceae provide genera, such as *Sphaerocoryn, Annickia*, *Petasites,* and *Sonchus,* which could be further investigated for their antimalarial effects, based on the in vitro and in vivo experimental results, and the generation of potential lead compounds for drug development. Whether they have an appropriate cytotoxicity profile and whether they will have low IC_50_ values in vivo should be investigated with the use of hyphenated techniques combined with omics-based approaches.

## Figures and Tables

**Table 1:** Selection of promising enriched extracts against *P. falciparum in vitro*. Chloroquine-resistant strains: W2, K1, W2, Cam WT_C580Y, and IPC4912. Chloroquine-sensitive strains: 3D7 and NF54.

Plant Species	Plant Parts	Type of Extract	IC_50_ Value against *P. falciparum* (μg/mL)	References
*Azadirachta indica* (A. Juss)	Leaves	Ethanol | Aqueous	7.4 (ethanol) and 8.6 (aqueous)	[57]
*Homalolepis suffruticosa* (Engl.) (Devecchi and Pirani)	Roots	Methanol	1.88 (W2)	[58]
*Goniothalamus* *Lanceolatus* (Miq.)	Roots	Methanol	2.7 (3D7) and 1.7 (K1)	[59]
*Vitex negundo* (L.)	Leaves	Chloroform	7.21 (3D7) and 7.43 μg/mL (K1)	[60]
*Petasites japonicus* (Siebold and Zucc.) (Maxim)	Leaves	Ethanol (70%)	8.48 (3D7) and 7.83 (Dd2)	[61]
*Senna occidentalis* (L.) (Link)	Leaves and seeds	Methanol	12.19 (leaves) and 6.82 (seeds)	[62]
	Roots	Methanol	1.76 (3D7)	
*Nauclea orientalis* (L.)	Leaves	Methanol	3.91 (3D7)	[63]
*Alchornea cordifolia* (Schumach.) (Müll. Arg.)	Herbal	Aqueous	5.8 (NF54), 17.4 (Cam WT_C580Y), and 15.8 (IPC 4912)	[64]
*Helianthus annuus* (L.)	Roots and leaves	Ethanol	2.3 (3D7)	[65]
*Harungana* *madagascariensis* Lam. ex Poir.	Barks	Aqueous	6.16 (NF54)	[66]
*Pericopsis laxiflora* (Benth. ex Baker) (Meeuwen)	Leaves	Methanol	7.44 (K1)	[66]
*Pleiocarpa bicarpellate* (Stapf)	Roots	Dichloromethane/Methanol (1:1)	3.5 (NF54)	[67]
*Psychotria apoda* (Steyerm.) (Delprete and J.H.Kirkbr.)	Leaves	Acid-base	>90% (W2)	[68]
*Andrographis paniculate* (Burm.f.) (Wall.)	Whole plant	Chloroform	6.36 (3D7) and 5.24 (K1)	[69]
*Globba malaccensis* (Ridl.)	Rhizomes	Ethanol	1.50 (K1)	[70]
*Sanchus arvensis* L.	Leaves	Ethyl acetate	2.9 (3D7)	[71]
*Terminalia arjuna* (Roxb. Ex DC.) (Wight and Arn.)	Fruits	Aqueous	4.05 (K1)	[72]
*Terminalia bentzoe* (L.) *L.f.*	Leaves	Methanol	6.06 (Dd2)	[73]
*Bridelia atroviridis Müll. Arg.*	Bark	Hydroethanolic	8.08 (Dd2)	[74]
*Annickia affinis* (Exell) (Versteegh and Sosef)	Leaves	Aqueous	1.49 (3D7)	[75]

## Data Availability

Not applicable.
